# Cellular Responses to Membrane and Nucleocapsid Viral Proteins Are Also Boosted After SARS-CoV-2 Spike mRNA Vaccination in Individuals With Either Past Infection or Cross-Reactivity

**DOI:** 10.3389/fmicb.2021.812729

**Published:** 2022-02-11

**Authors:** Alejandro Vallejo, Adrián Martín-Hondarza, Sandra Gómez, Héctor Velasco, Pilar Vizcarra, Johannes Haemmerle, José L. Casado

**Affiliations:** ^1^Laboratory of Immunovirology, Health Research Institute Ramón y Cajal (IRyCIS), University Hospital Ramón y Cajal, Madrid, Spain; ^2^Department of Infectious Diseases, Health Research Institute Ramón y Cajal (IRyCIS), University Hospital Ramón y Cajal, Madrid, Spain; ^3^Department of Prevention of Occupational Risks, Health Research Institute Ramón y Cajal (IRyCIS), University Hospital Ramón y Cajal, Madrid, Spain

**Keywords:** SARS-CoV-2, cellular immune response, epitope, cross-reactivity, COVID-19, mRNA vaccine

## Abstract

SARS-CoV-2 spike mRNA vaccines have shown remarkable clinical efficacy in the general population, although the nature of T-cell priming is not fully understood. We performed longitudinal spike-, membrane-, and nucleocapsid-specific T-cell analysis in individuals with past infection and infection-naïve individuals with cross-reactivity. We found an additional enhancement of T-cell response to the structural membrane (M) and nucleocapsid (N) SARS-CoV-2 proteins after mRNA vaccine in these individuals. Thus, despite the spike-specific response, we found that the first dose of the vaccine boosted a significant CD8 cell response to M and N proteins, whereas no cellular response to those proteins was found in infection-naïve individuals without pre-existing cross-reactivity who were tested for eventual asymptomatic infection. These findings highlight the additional benefit of mRNA vaccines as broad boosters of cellular responses to different viral epitopes in these individuals and suggest extended protection to other viral variants.

## Introduction

In COVID-19 patients, virus-specific CD4 and CD8 T-cell responses have a role in protective immunity against SARS-CoV-2 (Liu et al., 2020; Casado et al., 2021a). Thus, in convalescent individuals, a broad diversity of T-cell epitopes against viral structural and non-structural proteins have been reported (Kared et al., 2020; Peng et al., 2020), and a large majority are seemingly unaffected by current variants of concern (Tarke et al., 2021a). Additionally, there is solid evidence for the existence of cellular cross-reactivity to SARS-CoV-2 in 30–60% of unexposed individuals (Grifoni et al., 2020; Le Bert et al., 2020), showing also diversity of T-cell epitopes against viral proteins. Indeed, the sequence identity of the membrane (M) viral protein among common coronavirus and SARS-CoV-2 is much higher than for the spike (S) protein and receptor-binding domain (RBD), suggesting the potential of M protein as a target for cross-reactive T cells (Neuman et al., 2011). Also, the nucleocapsid (N) protein is the most abundant viral protein and is highly immunogenic during common coronavirus infections and may help to broaden the T-cell response and improve cross-protection (Vabret et al., 2020).

The Comirnaty mRNA vaccine encodes the full length of the S protein of SARS-CoV-2. To date, successful immune responses have been reported for mRNA vaccines against the SARS-CoV-2 S protein, with excellent humoral and cellular responses in patients with previous infection or pre-existing T-cell immunity (Sahin et al., 2020; Casado et al., 2021b). However, the details of response following SARS-CoV-2 vaccination in subjects with such pre-existing memory T cells are incompletely understood, and questions remain about the possibility of enhanced T-cell response to other immunodominant epitopes, as CD8 T cells of COVID-19 patients recognize other epitopes in SARS-CoV-2 that reside outside the S protein. Furthermore, it is necessary to determine the changes in the T-cell immunodominance induced by vaccination, and its differences with the protective ability of the multi-specific T-cell response induced physiologically by the natural infection, to clearly ascertain the level of protection achieved with vaccination.

To determine whether the Comirnaty mRNA vaccine, designed against epitopes included in the S protein, elicits cellular responses to other viral epitopes such as those included in the M and N proteins, we studied 56 health care workers (HCWs) previously included in other studies for post-vaccine humoral and cellular responses.

## Results

**FIGURE 1:**
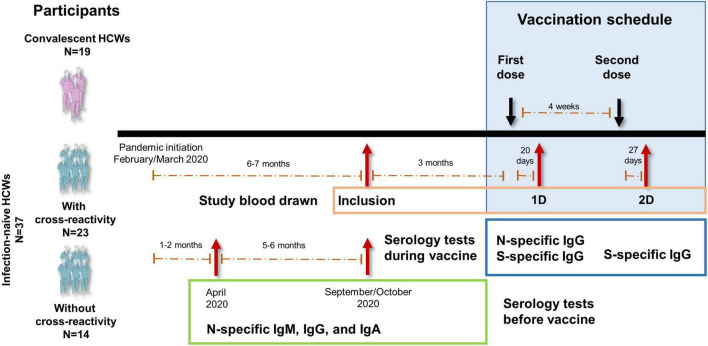
Study design diagram of the vaccination with Comirnaty (SARS-CoV-2 spike mRNA vaccine) to health care workers (HCWs). Infection-naïve HCWs with (*N* = 23) or without (*N* = 14) cross-reactivity and convalescent (*N* = 19) HCWs were included. Blood drawn time at inclusion and after the two doses of vaccine (vaccination schedule) are shown. Specific serology tests performed during the vaccination schedule and at and before inclusion from the beginning of the pandemic are also shown. All participants had been tested for clinical symptoms since the beginning of the pandemic and throughout the study.

Fifty-six HCWs were analyzed for the presence of cellular responses to SARS-CoV-2 for a median of 14 weeks before undergoing analytical determinations after the first and second doses of the mRNA vaccine (see Figure 1 for study design). Baseline characteristics of these individuals at inclusion are shown in Table 1. No significant differences in age, sex, or comorbidities between the groups were found.

**TABLE 1:** Characteristic of the 56 health care workers (HCW) included in the study.

	Convalescent HCW	Infection-naïve HCW with cross cellular reactivity	Infection-naïve HCW without cross cellular reactivity	ANOVA
	
	*N* = 19	*N* = 23	*N* = 14	*p*
Age (years)	57 [41–61]	52 [44–59]	53 [43–57]	0.717
Sex (female n, %)	11 (58%)	17 (74%)	9 (64%)	0.559
Body mass index (kg/m^2^)	24 [22–26]	23 [20–25]	23 [22–28]	0.215
Comorbidities (n, %):				
Hypertension	2 (10%)	4 (17%)	1 (7%)	0.358
Diabetes	0	2 (9%)	0	0.674
Before inclusion (n, %)*:				
SARS-CoV-2 RT-PCR**	15 (79%)	-	-	
SARS-CoV-2 IgM/IgG/IgA antibodies	19 (100%)	0	0	
SARS-CoV-2-compatible clinical symptoms	17 (89%)	0	0	
Time from infection to first dose (months)	10 [9–10]	-	-	
Time from inclusion to first dose (weeks)	14 [13–16]	14 [11–15]	14 [12–16]	0.722
N-specific SARS-CoV-2 IgG, antibodies (n, %):				
At inclusion***	11 (58%)	0	0	
After first dose (1D)	9 (48%)	0	0	
CD8 response to SARS-CoV-2 Spike at inclusion (n, %)	13 (68%)	9 (39%)	0	
CD4 response to SARS-CoV-2 Spike at inclusion (n, %)	12 (63%)	11 (48%)	0	

*See Figure 1 for specific time frame; **specific RT-PCR not performed to asymptomatic individuals (two individuals with clinical symptoms were not tested for RT-PCR);

***eight individuals lost antibodies titers.

**FIGURE 2:**
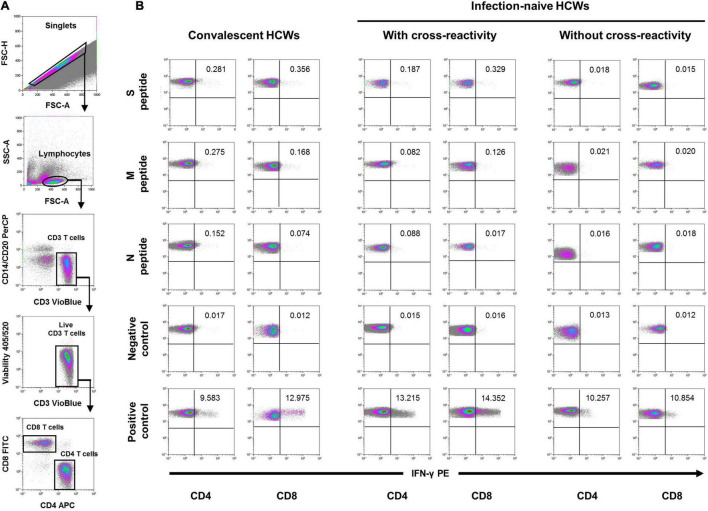
Flow cytometry strategy. **(A)** After gating of singlet cells (FSC-A/FSC-H density plot), lymphocytes were morphologically selected with FSC-A/SSC-A density plot, and then CD3 T cells were gated. Cell debris, monocytes, and B cells were excluded from the analysis with CD14- and CD20-PerCP antibodies, and live CD3 T cells were selected. IFN-γ expression was finally analyzed separately for CD4+ and CD8+ T cells and analyzed under five different conditions. **(B)** Stimulated with the three different SARS-CoV-2 peptides (S, M, and N peptides), unstimulated (negative control), and SEB-stimulated (positive control). A representative sample of each group at inclusion is shown for the five conditions in either CD4 or CD8 T cells. Percentage of cells expressing IFN-γ are also shown in each density plot.

**FIGURE 3:**
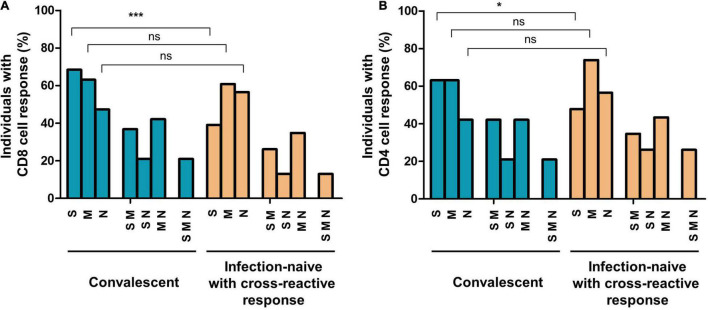
Proportion of individuals with cellular responses to SARS-CoV-2 S, M, and N proteins and different combinations in convalescents and infection-naïve individuals with cross-reactive responses at inclusion of the study (before initiation of vaccination), in CD8 **(A)** and CD4 **(B)** T cells. S, M, or N corresponded to subjects responsive to at least these proteins, including double and triple responders; SM, SN, or MN corresponded to double responders, including triple responders; SMN corresponded exclusively to triple responders. Ns, not significant; *p < 0.05; ***p < 0.001.

**FIGURE 4:**
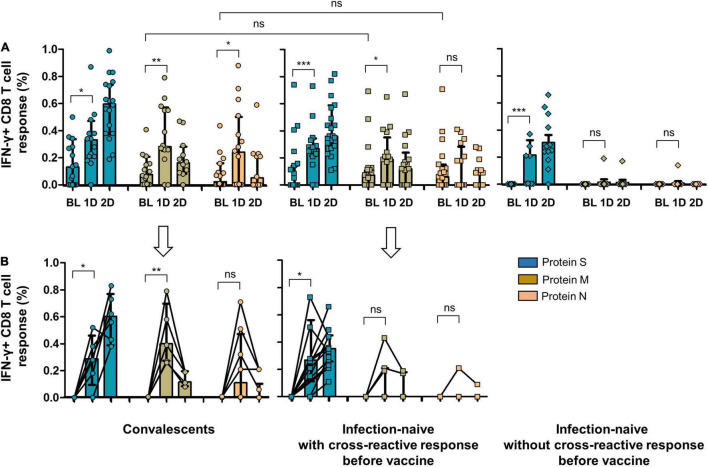
**(A)** CD8 cell response to SARS-CoV-2 S, M, and N proteins in convalescents, infection-naïve individuals with cross-reactive response, and infection-naïve individuals without cross-reactive response at inclusion of the study and after vaccine. **(B)** CD8 cell response to SARS-CoV-2 S, M, and N proteins after vaccine including only individuals without cellular response to S, M, or N peptides before vaccine (six convalescents, six infection-naïve individuals with cross-reactive response, and nine infection-naïve individuals without cross-reactive response). BL, baseline (inclusion); 1D, after first dose; 2D, after second dose; Ns, not significant; *p < 0.05; **p < 0.01; ***p < 0.001.

**FIGURE 5:**
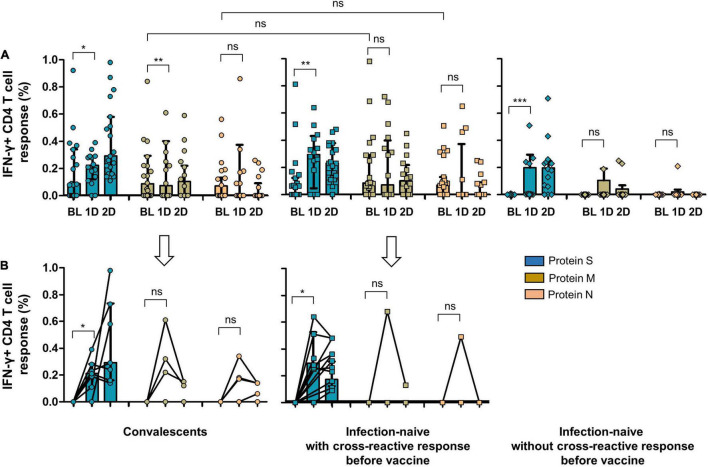
**(A)** CD4 cell response to SARS-CoV-2 S, M, and N proteins in convalescents, infection-naïve individuals with cross-reactive response, and infection-naïve individuals without cross-reactive response at inclusion of the study and after vaccine. **(B)** CD4 cell response to SARS-CoV-2 S, M, and N proteins after vaccine including only individuals without cellular response to S, M, or N peptides before vaccine (6 convalescents, 7 infection-naïve with cross-reactive response, and 11 infection-naïve without cross-reactive response). BL, baseline (inclusion); 1D, after first dose; 2D, after second dose; Ns, not significant; *p < 0.05; **p < 0.01; ***p < 0.001.

Analyzing the frequencies of subjects responsive to different virus peptides combinations (see Figure 2 for flow cytometry strategy), i.e., subjects responsive to at least S, M, or N peptides (including double and triple responders), at least double responders (including triple responders), and exclusively triple responders, we found that at inclusion, convalescent individuals showed similar frequencies of CD8 cell responses to M (63%) and N (47%) proteins compared to infection-naïve individuals with cross-reactive responses (60 and 56%, respectively). In parallel, similar CD4 cell responses to M and N proteins were found in convalescents (63 and 42%, respectively) compared to that found in infection-naïve individuals with cross-reactive responses (73 and 56%, respectively), as shown in Figures 3A,B. Furthermore, no statistical differences were found in the magnitude of cellular response to M or N proteins according to past infection (convalescents) or infection-naive individuals with cross-reactive cellular immunity, either in CD8 (*p* = 0.618 and *p* = 0.750, respectively) or CD4 (*p* = 0.591 and *p* = 0.636, respectively) cell responses (Figures 4, 5, respectively).

As expected, convalescents showed significant cellular reactivity against the S protein after vaccination compared to infection-naïve individuals with or without cross-reactivity, as shown in Figures 4, 5. It is worthy of note that after the first dose of the vaccine, we found a significant increase in the magnitude of CD8 cell response to M protein (*p* = 0.028), both in convalescent and individuals with a cross-reactive response (*p* = 0.043) and to N protein (*p* = 0.035) in those with past infection (Figure 4A). Moreover, when we selected only individuals without CD8 or CD4 cell responses to S, M, and N proteins at inclusion (6 convalescents, 6 infection-naïve individuals with cross-reactive response, and 9 infection-naïve individuals without cross-reactive response for CD8 cell responses, and 6 convalescents, 7 infection-naïve individuals with cross-reactive response, and 11 infection-naïve individuals without cross-reactive response for CD4 cell responses; Figures 4B, 5B, respectively), we also found an increment of cellular responses to M and N proteins (regardless of the expected increment of the cellular response to S proteins), although it was significant only in convalescents and to M protein (*p* = 0.004) after the first dose. It is worthy of note that the magnitude of the cellular responses to each epitope slightly decreased after the second dose.

**FIGURE 6:**
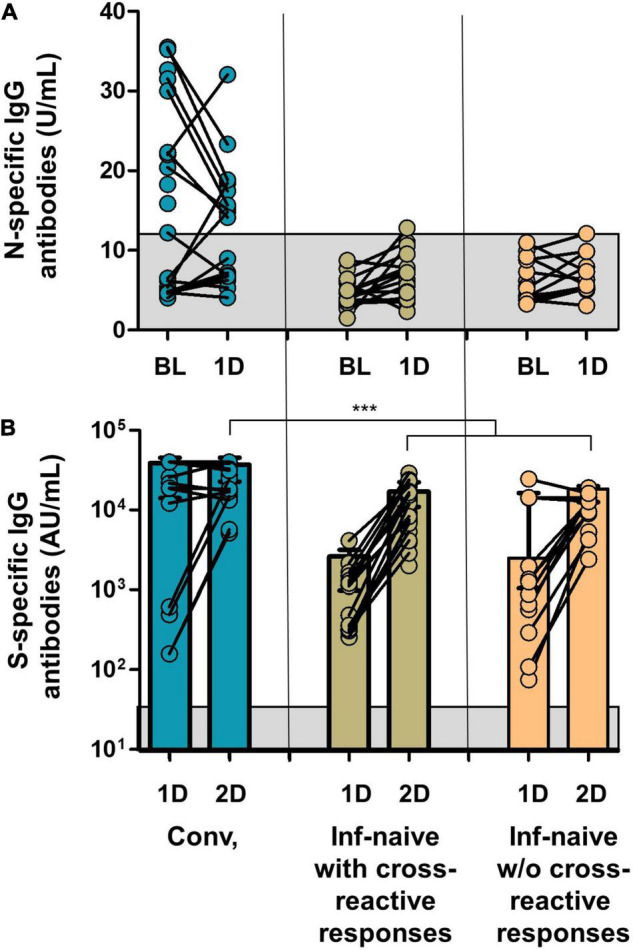
**(A)** SARS-COV-2 N-specific IgG antibodies in the groups of the study at inclusion and after the first dose of the vaccine. **(B)** SARS-CoV-2 S-specific IgG antibodies after the first and second doses. Gray squares show the limit of detection of the antibody tests. Conv., convalescents; ***p < 0.001.

Since vaccination aimed to boost spike-specific T-cell responses, we performed several strategies to clarify this unexpected extended response to epitopes other than S protein. First, we evaluated the cellular responses to the different viral proteins in infection-naïve individuals without cross-reactive responses at inclusion. Notably, none of these control individuals showed cellular responses after vaccination except for one participant who developed a CD8 cell response to M protein after the first and second doses and to N protein after the first dose, suggesting a recent infection (Figure 4A). Indeed, to rule out the eventual undiagnosed SARS-CoV-2 infection, we determined the specific antibodies against S protein (vaccination) but also against N protein (eventual asymptomatic infection). This N-specific IgG determination also revealed no new infections in convalescent or infection-naïve individuals with or without cross-reactive responses, except for the previously mentioned case (Figure 6).

**FIGURE 7:**
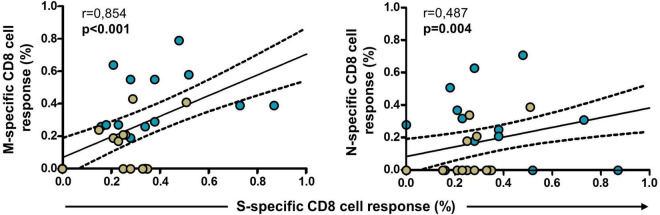
Correlation of S-specific CD8 cell response with M- and N-specific CD8 cell responses in convalescents (blue dots, *N* = 19) and infection-naïve individuals with cross-reactive response (green dots, *N* = 23) after the first dose of the vaccine. Significant when *p* < 0.05.

Finally, we analyzed the evolution of the responses to S, M, and N proteins after vaccination. Thus, CD8 cell response to M and N proteins in convalescents and infection-naïve with cross-reactive response correlated with CD8 cell response to S protein after the first dose (*p* < 0.001 and *p* = 0.004, respectively), suggesting a close relationship between the vaccine and the magnitude of the cellular response to M and N proteins, as shown in Figure 7. In this analysis, individuals without responses to M or N proteins after the first dose were also included.

## Discussion

In this study, we demonstrated that SARS-CoV-2 mRNA vaccines, encoding the full-length S protein, might trigger the cellular response to other viral proteins in individuals with past infection or infection-naïve individuals with previous cross-reactivity, although the contribution of these responses to the control of infection is still under investigation. The presence of cross-reactive responses in infection-naïve individuals has been documented in numerous studies. Le Bert et al. found specific cellular responses in uninfected individuals mainly to structural and non-structural proteins, measured by the production of IFN-γ and TNF-α by CD4 and CD8 T cells (Le Bert et al., 2020). Grifoni et al. found that 40–60% of unexposed individuals had SARS-CoV-2-reactive CD4 T cells (Grifoni et al., 2020). Other studies described that pre-existing cross-reactive memory T cells found in some uninfected HCWs (despite being exposed to SARS-CoV-2), as described in pre-pandemic samples, may lead to abortive seronegative infections (Shimizu et al., 2021; Flemming, 2022). This cross-reactivity is likely to be induced by seasonal coronaviruses since they present high sequence conservation. Although immune responses to these seasonal coronaviruses seem to decline rapidly, eventual reinfection could occur repeatedly within a single year, boosting the cross-reactive immunity.

To date, it is well-established that convalescent individuals harbor polyfunctional SARS-CoV-2-specific T cells that display a stem-like memory phenotype (Sekine et al., 2020; Weiskopf et al., 2020). After vaccination, both CD4 and CD8 T-cell responses are dominated by central memory-like cells, similar to memory T cells generated following natural infection (Painter et al., 2021). Thus, a diverse array of CD8 T-cell responses to SARS-CoV-2 antigens are likely to be present in both convalescent individuals and as a consequence of previous infections to seasonal coronaviruses in naïve individuals (Lee et al., 2020). These CD8 T cells are effector and central memory cells with functional potential upon antigen re-exposure (Peng et al., 2020), as we previously reported (Casado et al., 2021a).

However, unexpectedly, the cellular response was not exclusively directed toward S-related epitopes after spike mRNA vaccination. It is still controversial how exclusively specific T-cell antigen recognition is (Petrova et al., 2012), and reports are suggesting that T-cell recognition might be highly promiscuous with individual T-cell clones being able to cross-reactively recognize different epitopes (Wooldridge et al., 2012). In COVID-19, most human leukocyte antigen (HLA) alleles are associated with multiple epitopes, and the same epitope may be presented by multiple HLA alleles, as evidenced by studies of the related SARS-CoV-2, as well as other viruses (Quadeer et al., 2021). Indeed, we found a significant response to M protein in convalescent individuals who did not show or had an undetectable M-specific response before vaccination, marking the role of central memory CD8 T cells and the possibility of being activated after an antigenic stimulus. Also, mRNA vaccines have been shown to produce non-specific antigen stimulation of type I interferon pathways, as demonstrated with other formulations of mRNA in animal models (Sadarangani et al., 2021).

This unexpected finding has not previously been discussed in-depth because, in most studies, cellular responses were assessed by CD4 and CD8 T-cell assays after activation either with pooled spike peptides or with a mega-pool of SARS-CoV-2 peptides. In a recent study similar to ours, unexpected T-cell responses to N protein after vaccination in convalescent individuals were found, although it was not specifically interpreted (Loyal et al., 2021; Lucas et al., 2021; Zollner et al., 2021).

As shown previously, we found a reduction in the cellular response to the different epitopes between the first and second doses for the different CD8 T cells. It has been demonstrated that SARS-CoV-2-recovered individuals had maximal CD4 and CD8 cell responses following the first dose of mRNA vaccine, and there was little additional T-cell boosting after the second dose (Painter et al., 2021; Samanovic et al., 2021). Whether this retraction was due to T cells addressed to S protein or other SARS-CoV-2 proteins is of interest since most studies of cellular responses analyzed were restricted to S-specific or mega-pool proteins. Thus, we found that CD8 T cells that target different epitopes such as M and N proteins are substantially induced early after a first dose of vaccine, and subsequent boost vaccinations did not further increase the magnitude of the response to M protein.

The possibility that mRNA vaccines encoding the full-length S protein trigger an extended response to other viral proteins could be of special relevance. These re-activated extended memory CD8 T cells are likely to be less impacted by antibody escape mutations in variant viral strains, as T cells can recognize different peptide epitopes distributed throughout the SARS-CoV-2 proteins (Lucas et al., 2021; Tarke et al., 2021b). After natural infection, there is evidence that the development of immunity requires recognition of multiple SARS-CoV-2 epitopes, and in other viral infections, a multi-specific T-cell response appears to be an important determinant of viral clearance (Bertoletti et al., 2021). However, it might be possible that despite this broad T-cell response observed in SARS-CoV-2-recovered individuals, robust T cells specific for a single protein can be equally protective. One of the mechanisms involved in this phenomenon is that strong immune activation, such as that produced after vaccination, could drive the development or exacerbation of non-vaccine-related immune responses (Bergamaschi et al., 2021). Then, bystander activation after spike-specific vaccination could activate unrelated memory T cells, such as M- or N-specific cells that are already present in convalescent and infection-naïve individuals with cross-reactivity.

Our study has several limitations in addition to the limited number of individuals included. Despite repeatedly negative serological tests, we cannot exclude the possibility of SARS-CoV-2 past infection in some infection-naïve individuals with a cross-reactive response.

In conclusion, our data suggest that a wide vaccine-derived T-cell response could trigger, at least in part, a similar memory T-cell response to that generated following SARS-CoV-2 infection in either convalescent or infection-naïve individuals with cross-reactivity. This could explain the rapid cellular response observed in these individuals following the first dose of SARS-CoV-2 spike mRNA vaccine that may contribute to induce adequate protection against severe COVID-19 (Cavanaugh et al., 2021). Comparison of outcomes post-vaccination considering also non-spike responses will add light to our understanding on the role of the extended T-cell response to different viral peptides in the clinical evolution of convalescent individuals.

## Materials and Methods

### Ethics Statements

The initial cross-sectional study and its amendments were approved by the Ethics Committee of the University Hospital Ramon y Cajal, Madrid (EC number 162/20), and performed following the ethical guidelines. Written informed consent was obtained from all participants in accordance with the Declaration of Helsinki.

### Study Participants

This longitudinal study included 56 Caucasian HCWs who completed a two-dose vaccination regimen with Comirnaty (SARS-CoV-2 spike mRNA vaccine, Pfizer-BioNtech). Nineteen were convalescents with reported positive PCR tests and/or anti-SARS-CoV-2 antibodies (N-specific IgG, IgM, and/or IgA antibodies). Thirty-seven were SARS-CoV-2-naïve individuals without having had any clinical symptomatology compatible for COVID-19 since the beginning of the pandemic (February/March 2020) and with continued serological negativity results since then, including one serologic survey 1.2 [1.0–1.9] months after the beginning of the pandemic and the survey performed at inclusion (5.2 [4.9–5.6] months after the first survey). The study design and the time points for the different serological surveys are shown in Figure 1. Among these infection-naïve individuals, 23 had cellular response (cross-reactive response) to any of the structural SARS-CoV-2 viral proteins (S, M, or N proteins) either in CD8 or CD4 T cells, and 14 had neither CD4- nor CD8-specific responses to any of the SARS-CoV-2 proteins (S, M, or N proteins).

### Humoral Response to the SARS-CoV-2 mRNA Vaccine

The participants were tested for SARS-CoV-2 spike-specific IgG antibodies (SARS-CoV-2 IgG II Quant, Abbott, Maidenhead, United Kingdom) after the first and second doses of the vaccine. This test had a threshold of 50 arbitrary units per milliliter (AU/ml), as specified by the manufacturer. Participants were also tested for SARS-CoV-2 N-specific IgG antibodies (COVID-19-SARS-CoV-2 IgG ELISA, Demeditech, Germany) before vaccination and after the first dose of the vaccine to confirm incident SARS-CoV-2 infections during the study. Results were recorded as relative units per milliliter (U/ml), with a threshold of 11 U/ml, as specified by the manufacturer.

### IFN-γ-Producing CD8 and CD4 T Cells

Participants were tested for the presence of IFN-γ-producing CD8 and CD4 T cells after *in vitro* stimulation of lymphocytes with SARS-Cov-2 S, M, and N peptides at inclusion and after both doses of the vaccine. Briefly, after centrifugation at 200 *g* for 10 min, plasma fraction was collected and again centrifuged at 1,200 *g* for 15 min, aliquoted, and stored at −80°C. The cellular fraction was diluted with phosphate-buffered saline (PBS) and subjected to Ficoll density gradient centrifugation at 500 *g* for 20 min. Peripheral blood mononuclear cells (PBMCs) were washed and frozen in fetal bovine serum (FBS) with 8% dimethyl sulfoxide (DMSO, Sigma, St. Louis, MO, United States) in liquid nitrogen. PBMCs were thawed and plated in 96-well flat-bottom plates at 10^6^ cells/well in RPMI-1640 culture medium (Gibco, Dublin, Ireland) supplemented with 10% human serum (AB serum, Sigma, St. Louis, MO, United States), 100 IU of penicillin/streptomycin/ml (Gibco, Dublin, Ireland), and 2 mM L-glutamine, and after 24 h cells were stimulated in five different conditions in the presence of 1 μg/ml of purified anti-CD28 antibody (Miltenyi, Bergisch Gladbach, Germany). Three wells were stimulated with each of the SARS-CoV-2 peptide pools S, M, and N at a concentration of 1 μg/ml. Each peptide pool was composed of 15-mer sequences with 11 amino acids overlap, covering the immunodominant sequence domains of the S glycoprotein, the complete sequence of the M glycoprotein, and the complete N phosphoprotein of SARS-CoV-2 (PepTivator SARS-CoV-2 Prot S, M, and N, Miltenyi-Biotec, Cologne, Germany). In addition, one well was assayed with culture medium alone as a negative control (unstimulated), and another well was stimulated adding 1.5 mg of SEB (staphylococcal enterotoxin B, Sigma, St. Louis, MO, United States) as the positive control. An unresponsive sample to SEB would be excluded from the analysis. Stimulated PBMCs were incubated for 2 h before adding brefeldin A (Rapid Cytokine Inspector CD4/CD8 T-cell kit, Miltenyi, Bergisch Gladbach, Germany) into the medium to stop cytokine release and kept in culture for another 12 h. After stimulation, staining of the cells was carried out with the following fluorochromes-conjugated antibodies using a Rapid Cytokine Inspector CD4/CD8 T-cell kit (Miltenyi, Bergisch Gladbach, Germany): CD3-VioBlue, CD4-APC, CD8-FITC, CD14-PerCP, CD20-PerCP, IFN-γ-PE, and FcR blocking reagent. To exclude dead cells, viability 405/520 fixable dye staining (Milteny, Germany) was added for the last 10 min of incubation. Fixation and permeabilization were performed according to the manufacturer’s protocol. Samples were measured and analyzed by flow cytometry on a MACSQuant Analyzer 10 using MACSQuantify software with the following strategy: Single (FSC-A/FSC-H density plot) cells were first selected. Then, lymphocytes were morphologically selected followed by the exclusion of cell debris, monocytes, and B cells with CD14- and CD20-PerCP antibodies. Then, live CD3 T cells were gated and a minimum of 5 10^4^ CD4 and CD8 T cells were analyzed separately for the expression of IFN-γ under five different conditions, including the stimulation with SARS-CoV-2 peptides, unstimulated (negative control), and SEB-stimulated (positive control) cells (see Figure 2 for flow cytometry strategy). The frequency of cell response was calculated by subtracting twofold the background observed in the absence of stimulation from the frequency observed in the presence of viral peptides.

### Statistical Analysis

Comparisons between groups were performed using two-tailed statistical tests, chi-square or Fisher’s exact tests for categorical variables, and Mann–Whitney test or one-way analysis of variance (Kruskal–Wallis test) with Dunn’s correction for multiple comparisons, as appropriate. Paired samples were compared using the Wilcoxon-signed rank test. Correlation analyses were performed using the non-parametric Spearman test. Statistical significance was defined as two-sided *p*-values below 0.05.

## Data Availability Statement

The raw data supporting the conclusions of this article will be made available by the authors, without undue reservation.

## Ethics Statement

The studies involving human participants were reviewed and approved by Ethics Committee of the University Hospital Ramon y Cajal, Madrid, Spain (EC number 162/20). The patients/participants provided their written informed consent to participate in this study.

## Author Contributions

JC and AV conceived and designed the study, analyzed the results, and wrote the manuscript. HV conducted the study, followed up participants, collected data, and performed analytical determinations. PV and JH conducted the study, collected data, and analyzed the results. All the authors revised the manuscript and approved the final version.

## Conflict of Interest

The authors declare that the research was conducted in the absence of any commercial or financial relationships that could be construed as a potential conflict of interest.

## Publisher’s Note

All claims expressed in this article are solely those of the authors and do not necessarily represent those of their affiliated organizations, or those of the publisher, the editors and the reviewers. Any product that may be evaluated in this article, or claim that may be made by its manufacturer, is not guaranteed or endorsed by the publisher.
